# Incidence, prevalence and associated factors of mother-to-child transmission of HIV, among children exposed to maternal HIV, in Belgaum district, Karnataka, India

**DOI:** 10.1186/s12889-019-6707-3

**Published:** 2019-04-06

**Authors:** Rajaram Subramanian Potty, Anju Sinha, Rajeev Sethumadhavan, Shajy Isac, Reynold Washington

**Affiliations:** 1 Karnataka Health Promotion Trust (KHPT), IT Park, Rajajinagar Industrial Area, Behind KSSIDC Admin. Office, Rajajinagar, Bengaluru, 560044 India; 2 Indian Council of Medical Research (ICMR) Headquarters, Ansari Nagar, New Delhi India; 3 St John’s Research Institute, Bengaluru, India; 4 Department of Community Health Sciences, University of Manitoba, Winnipeg, Manitoba Canada

**Keywords:** HIV incidence; mother-to-child transmission, India, Breastfeeding, Prevalence, Nevirapine

## Abstract

**Background:**

India lacks data on the incidence of Paediatric HIV. In 2010, the Indian Council of Medical Research commissioned a task force study to estimate the paediatric HIV burden in Belgaum district, Karnataka, India. We estimated the HIV incidence, prevalence and associated risk factors of mother to child transmission of HIV among children exposed to maternal HIV by age 24 months.

**Methods:**

We included Belgaum resident pregnant women who tested HIV positive between January 1st, 2011 and May 31st, 2013 and who provided consent. Their babies were tested for HIV at three time intervals using DNA PCR dry blood spot (DBS) method at 6–10 weeks and 6–9 months, and using Antibody tests at 18–24 months of age. We estimated cumulative incidence using survival analysis that considered censoring of cases and prevalence rates of HIV by age 24 months. Using competing-risk survival regression model, we examined the correlates of transmission of HIV among babies exposed to maternal HIV.

**Results:**

Among 487 children of HIV positive mothers recruited in the study, the cumulative incidence rate by 24 months of age was 4.8 per 1000 person months [95% CI: 3.5–6.6]. The HIV prevalence rate among babies exposed to maternal HIV until 24 months was 7.8% [95% CI: 5.7–10.7]. Mother’s age above 30 years, and breastfeeding duration of more than six months were factors that significantly increased the HIV transmission; adjusted hazard ratio (AHR) 6.98 [95% CI: 1.73–28.16] and 5.28 [95% CI, 1.75–15.90], respectively. The risk of MTCT was significantly reduced if both mother and baby had received Nevirapine at delivery [AHR 0.25; 95%CI: 0.10–0.61] and if either mother or baby had been given Nevirapine at delivery [AHR 0.12; 95%CI: 0.03–0.49].

**Conclusion:**

The study findings suggest that mother’s age above 30 years and breastfeeding beyond 26 weeks is associated with higher rates of HIV transmission from mother to child. It confirms the benefits of providing anti-retrovirals (Nevirapine) in reducing mother to child transmission of HIV. Effective strategies to promote safe infant feeding practices, including avoidance of mixed feeding beyond 26 weeks among HIV infected mothers, is critical to reduce incidence of paediatric HIV in India.

## Background

Mother to child transmission remains the major source of paediatric human immunodeficiency virus (HIV) infections in India, accounting for more than 12% of all new infections [1]. HIV infection in children can occur during pregnancy, delivery or through breastfeeding [2]. The prevention of parent to child transmission (PPTCT) program as it is referred to in India, plays a critical role in reducing the incidence of HIV among children. Following the landmark research study on prevention of vertical HIV transmission by the AIDS Clinical Trial Group (ACTG-076) in 1994 [3], there have been significant efforts in India to scale up the coverage of HIV testing and counselling, to prevent linkage loss for treatment initiation for those mothers detected HIV infected and to ensure early infant diagnosis and initiation of treatment for infants detected to have HIV [2]. In 2012, the World Health Organization (WHO) announced a global plan to eliminate mother-to-child transmission (MTCT) by 2015 [4].

The PPTCT interventions in India, under the aegis of the National AIDS Control Organisation (NACO), have evolved over the years. The PPTCT initiative began with HIV testing and counselling for pregnant women and the use of single dose Nevirapine to HIV infected women and exposed babies in 2002 [2]. The program launched early infant diagnosis (EID) of HIV among HIV-exposed infants and children under 18 months of age in 2010 [5]. Based on the WHO (2010) guidelines, in September 2012, NACO rolled out the triple drug Anti-retroviral Treatment (ART) (option B) in the three southern high prevalence states of Andhra Pradesh, Telangana and Karnataka initially and subsequently in Tamil Nadu [6]. Following the new WHO (2013) guidelines (called Option B+), NACO initiated the provision of life-long (triple drug regimen) Anti-retroviral treatment (ART) to all pregnant and breastfeeding women living with HIV effective from January 2014 [2].

Evidence suggests that various factors affect the vertical transmission of HIV. In the absence of any intervention, approximately one in three children born to HIV infected women identified will be infected with HIV in breastfeeding populations [7, 8]. Transmission rates of 1–2% have been reported in studies from developed countries. This low rate of HIV transmission is attributed to ART, caesarean section delivery, and avoidance of breastfeeding [9–12]. Transmission risk increases with low CD4 counts and high viral loads in the mother [13, 14]. In the era of highly effective ART, the role of an elective caesarean section to prevent MTCT appears to be limited [15].

In this study, we estimated the incidence, prevalence, and examined the role of associated factors of HIV infection, among children exposed to maternal HIV, in Belgaum district in Karnataka state in India. Few studies have tried to assess the risk of MTCT of HIV [16–19] in India. They were either confined to a single facility or assessed the risk among urban dwellers only. This district- wide study included both public and private sector health facilities, located in urban and rural settings.

Belgaum district is situated in the north-western part of Karnataka and is one of the four divisional headquarters of Karnataka. In 2011, the district had a population of 4,779,661 and is the second most populous district in the Karnataka state. The overall sex ratio was 973 females per 1000 males. Literacy rate among the population aged 7 and above was 74%: 83% and 65% among males and females, respectively. The proportion of population of children (0–14 years) was 29%, 10% were aged 60 years and over, and 61% were in the working age group (15–59 years). HIV prevalence among the general population aged 15–49 in 2010 in Belgaum district was 2.2% [20]. HIV sentinel surveillance (HSS) data for the year 2012 indicated a HIV prevalence of 0.75% among ANC attendees. HIV sentinel surveillance is restricted to data from two large urban-centric, public sector hospitals. These hospitals are also designated ART centres, and hence tend to have larger proportions of HIV infected mothers visiting these centres, as they get referred from lower level facilities, when detected with HIV. In the same year, the level of HIV positivity was lower at 0.21% among all PPTCT attendees [21]. The PPTCT attendees represent more than 90% of all pregnant women, including those resident in rural and urban areas and attending either private or public facilities.

## Methods

### Study design and participants

The cohort study enlisted, contacted and followed up every identified HIV infected pregnant woman and the HIV exposed child, who were residents in Belgaum district. A facility mapping was the first step to identify all urban as well as rural facilities that conducted HIV testing for pregnant women. We prepared a line list of all HIV infected pregnant women detected during 1 January, 2011 and 31 May, 2013 from all the health care facilities (HCFs), including Integrated Counselling and Testing centres (ICTC), PPTCT centres, anti-retroviral treatment (ART) centres, link ART centres, and private hospitals. From the list we excluded duplicates, non-residents of the district, non-traceable addresses, woman who were no longer pregnant, or had not delivered a baby in the last 3 months, and those who had migrated out of the district. These recruited HIV infected women and their babies were followed up until 30 Nov 2014 (closure of the study) or until the HIV Antibody test was completed for babies between the age of 18–24 months. Trained field investigators under supervision of field supervisors, collected information on socio-demographic characteristics, date of HIV testing of mother, date of start of ART, date and type of delivery, antiretroviral services received during delivery, sex of the infant, type and duration of breastfeeding and other details. In each follow-up visit, the mother was asked about whether she was still breast feeding or whether she had stopped breast feeding. If the mother reported that she had stopped breastfeeding, then the information on the number of weeks that she had breastfed, was collected. During the data analysis this was recoded and grouped as not breastfed, breastfed for 1–26 weeks and more than 26 weeks.

The field investigators were graduates, who received a two-week training on research ethics and the research protocols, before induction. They visited the recruited mothers in their homes and used a structured pre-tested questionnaire to collect the data. Data was captured at different times during the follow up, but mostly after delivery of the baby. The HIV test results of the babies were collected as per the national Early Infant Diagnosis (EID) protocol of NACO [22]. NACO recommends a HIV test three times. The first two tests use DNA PCR dry blood spot (DBS) method at 6–10 weeks and 6–9 months of age. The third test is an antibody test at 18–24 months of age. Trained lab technicians (LTs) or the designated ICTC in the district collected the blood samples from the HIV exposed children, at the specified ages. The LTs transported the blood samples to the designated testing centres, as per testing protocols and collected the results. The National Institute of Mental Health and Neurosciences (NIMHANS), Bengaluru conducted the DNA PCR tests on DBS samples. The ICTCs conducted the HIV antibody tests on the blood samples collected through venepuncture. The study team obtained the test results and entered these into the data base. Not all the children followed-up in the study could be tested all three times. This is because some of them died, or were recruited after the specified age, or were lost to follow up. Data entry operators transcribed paper based forms into a Microsoft Office Access database, after the supervisors had verified forms for completeness, timeliness and accuracy.

### Data analysis

Percentage distribution is used to describe the study population in relation to socio-demographic variables. We estimated the cumulative incidence rate based on the results of HIV testing carried out at different ages of the baby. We used survival analysis to estimate the cumulative incidence rate which is expressed per 1000 person months. We considered testing positive for HIV at the first test as the event and the age (calculated in months) at which the baby was either first identified as HIV positive, or age at which the baby was tested negative for the latest test, or age at which the baby died before testing positive, as the duration in the survival analysis. Further, we also calculated the HIV prevalence rate of MTCT, with respective confidence intervals at 95%. We provided the prevalence rate to understand the disease burden in the study population. Finally, we applied Cox proportional hazard model that considers competing risk to identify and determine the effect of each independent variable on the MTCT of HIV by the age 24 months, and to control for the effect of confounding. In the Cox proportional hazard model, we considered death as the competing event that impedes the occurrence of HIV infections. Results were expressed as hazard ratios with their 95% confidence intervals (CI). A *p*-value of less than 0.05 at 95% CI data was considered statistically significant. All analyses were performed using Stata software version 12.0 (Stata Corporation, College Station, Texas, USA).

### Ethical approval

Regulatory approvals for the study were obtained from the National AIDS Control Organisation (NACO) and the Karnataka State AIDS Prevention Society (KSAPS). Ethics approval was obtained from the Institutional Ethics Committee of St John’s Medical College and Research Institute, Bengaluru, India. We sought informed written consent from all the eligible women included in the final list before recruiting them for the study.

## Results

We recruited 469 HIV infected mothers who had delivered between 2011 and 2013, and who consented to participate in the cohort study. Twenty-seven mothers had a repeat pregnancy during the study period. Thus the total number of pregnancies was 496. Among the 496 pregnancies, 477 (96%) resulted in live births, 10 (2%) in abortions and 9 (2%) in still births. Among the live births, 10 were twin pregnancies. Thus, the total number of live born babies was 487. In total, 454 of these babies were tested for HIV infection at least once. The remaining 33 babies were never tested for HIV, either because they died before the first test (23 babies) or were lost to follow up (10). By the age of 24 months, we identified 38 babies as HIV positive. The median age at tests was 1.7 months for the first test, 6.3 months for the second test and 18.6 months for the third test. The median age at which children tested HIV positive was 6.3 months. During the course of the study 33 babies died, 10 among these babies were tested for HIV at least once before death, of which only one was found to be HIV positive. Among the babies who died, 16 babies died before 28 days of age, 15 babies died between 28 days and 1 year of age and the remaining 2 babies died after 1 year of age.

### Profile of the mother or the baby

Table 1 provides the percentage distribution of babies according to various characteristics of mother, sex of the baby, status of ART initiation by the mother, breastfeeding status and Nevirapine status. Most of the mothers were aged between 20 and 29 years (81%). About 21% of pregnant women knew that they were HIV positive before their current pregnancy. A quarter of the mothers were initiated on ART more than 90 days prior to the delivery, 17% were initiated the ART within 90 days prior to delivery and 9% were initiated on ART after their delivery. The remaining 48% had never been initiated on ART. However, 83% of mothers and babies received Nevirapine as ARV prophylaxis. In 12%, either mother or baby were given Nevirapine. In the remaining 5%, neither the mother nor the baby received Nevirapine. One-third of the babies were not breastfed at all. Half of the babies were breastfed for durations between 1 week and 26 weeks and the remaining 17% of the babies were breastfed for more than 26 weeks. The mode of delivery was vaginal for most of the pregnant women and 11% of babies were delivered through caesarean section.

**Table 1:** Percent distribution of live births according to selected characteristics

Characteristics	Number of live births	Percent
Age of mother at pregnancy		
< 20	23	4.7
20–24	204	41.9
25–29	191	39.2
30+	69	14.2
Education of mother		
Illiterate	169	34.7
Primary school	98	20.1
Above primary school	220	45.2
Working status of mother		
Not working	416	85.4
Working	71	14.6
Know HIV positive status prior to pregnancy		
No	384	79.2
Yes	101	20.8
Status of ART initiation by mother		
Not on ART	238	48.9
Started ART more than 90 days prior to delivery	121	24.9
Started ART within 90 days prior to delivery	83	17.0
Started ART after delivery	45	9.2
Sex of the child		
Boy	249	51.1
Girl	238	48.9
Status of Nevirapine administration		
Any one given Nevirapine	58	11.9
Both, mother and baby given Nevirapine	404	83.0
None given Nevirapine	25	5.1
Duration of breastfeeding		
Not breastfed	164	33.7
1–26 weeks	240	49.4
Above 26 weeks	82	16.9
Mode of delivery		
Vaginal	433	88.9
Caesarean	54	11.1
Total	487	100.0

### Cumulative incidence of MTCT

We estimated the cumulative incidence rate (CIR) of MTCT per 1000 person months by age 24 months and the results are presented in Table 2. Over all, the CIR by age 24 months was about 5 per 1000 person months. The CIR by age 24 months was found to be higher among babies of illiterate mothers (5.7 per 1000 person months), babies whose mother’s age was 30 years or more (8.1 per 1000 person months), and among those who knew their HIV status prior to pregnancy (5.3 per 1000 persons) as compared to mothers of their counterpart. According to the status of ART initiation, we noticed that the CIR was higher for mothers who were started the ART more than 90 days prior to delivery (7.4 per 1000 person months) and it was lower for mothers who were initiated ART after delivery (2.5 per 1000 person months). Babies who were not given Nevirapine (18.5 per 1000 person months) and who were breastfed for more than 26 weeks (6 months) were found to have higher CIR (7.7 per 1000 person months). Similarly, CIR by age 24 months was found to be higher for babies who were born vaginally (5.3 per 1000 person months) than if delivered by caesarean section (1.1 per 1000 person months).

**Table 2:** Cumulative incidence rate (per-1000 months) of vertical transmission of HIV among children by 24 months according to selected characteristics

Characteristics	Total person-months	Number of infected babies	Cumulative incidence rate	95% CI	
Age of mother at pregnancy					
< 20	345	1	2.90	0.41	20.58
20–24	3328	15	4.51	2.72	7.48
25–29	3213	14	4.36	2.58	7.36
30+	989	8	8.09	4.05	16.18
Education of mother					
Illiterate	2650	15	5.66	3.41	9.39
Primary school	1613	7	4.34	2.07	9.10
Above primary school	3612	16	4.43	2.71	7.23
Working status of mother					
Not working	6722	33	4.91	3.49	6.91
Working	1153	5	4.33	1.80	10.41
Know HIV positive status prior to pregnancy					
No	6153	29	4.71	3.28	6.78
Yes	1686	9	5.34	2.78	10.26
Status of ART initiation by mother					
Not on ART	3836	18	4.69	2.96	7.45
Started ART more than 90 days prior to delivery	1890	14	7.41	4.39	12.51
Started ART within 90 days prior to delivery	1362	4	2.94	1.10	7.82
Started ART after delivery	787	2	2.54	0.64	10.16
Sex of the child					
Boy	4036	22	5.45	3.59	8.28
Girl	3839	16	4.17	2.55	6.80
Status of Nevirapine administration					
Any one given Nevirapine	978	3	3.07	0.99	9.51
Both, mother and baby given Nevirapine	6573	29	4.41	3.07	6.35
None given Nevirapine	325	6	18.48	8.30	41.14
Duration of breastfeeding					
Not breastfed	2553	4	1.57	0.59	4.18
1–26 weeks	3864	23	5.95	3.96	8.96
Above 26 weeks	1438	11	7.65	4.24	13.81
Mode of delivery					
Vaginal	6946	37	5.33	3.86	7.35
Caesarean	930	1	1.08	0.15	7.64
Total	7875	38	4.83	3.51	6.63

### Prevalence of MTCT

From the CIR for the exposed babies by age 24 months, we calculated the HIV prevalence to understand the overall transmission rate of HIV infection in the study area and the results are presented in Table 3. Nearly, 7.8% (95%CI: 5.7–10.7) of the exposed babies were infected with HIV by age 24 months through vertical transmission. The rate of transmission was 24% (95%CI: 10.8–53.4) for babies when neither the baby nor the mother was administered Nevirapine. Similarly, the HIV prevalence was highest for babies who were breastfed for more than 26 weeks (13.4%; 95%CI: 7.4–24.2) as compared to non-breastfed babies (2.4%; 95%CI: 0.9–6.5). MTCT HIV prevalence was higher if the mother was initiated ART more than 90 days prior to delivery (11.6%; 95%CI: 6.9–19.5), and if the mother’s age at pregnancy was 30 years or more (11.6%; 95%CI: 5.8–23.2) as compared to other mothers. Boys (8.8%; 95%CI: 5.8–13.4) were found to have higher MTCT HIV prevalence than girls (6.7%: 95%CI: 4.1–11.0). MTCT HIV prevalence was found to be higher among babies delivered vaginally (8.6%; 95%CI: 6.2–11.8) as compared to babies born through caesarean section (1.9%; 95%CI: 0.3–13.1).

**Table 3:** HIV prevalence rate (per 100) among children by 24 months according to selected characteristics

Characteristics	Number of exposed babies	Number of infected babies	Prevalence rate	95% CI	
Age of mother at pregnancy					
< 20	23	1	4.35	0.61	30.87
20–24	204	15	7.35	4.43	12.20
25–29	191	14	7.33	4.34	12.38
30+	69	8	11.59	5.80	23.18
Education of mother					
Illiterate	169	15	8.88	5.35	14.72
Primary school	98	7	7.14	3.41	14.98
Above primary school	220	16	7.27	4.46	11.87
Working status of mother					
Not working	416	33	7.93	5.64	11.16
Working	71	5	7.04	2.93	16.92
Know HIV positive status prior to pregnancy					
No	384	29	7.55	5.25	10.87
Yes	101	9	8.91	4.64	17.13
Status of ART initiation by mother					
Not on ART	238	18	7.56	4.77	12.00
Started ART more than 90 days prior to delivery	121	14	11.57	6.85	19.54
Started ART within 90 days prior to delivery	83	4	4.82	1.81	12.84
Started ART after delivery	45	2	4.44	1.11	17.77
Sex of the child					
Boy	249	22	8.84	5.82	13.42
Girl	238	16	6.72	4.12	10.97
Status of Nevirapine administration					
Any one given Nevirapine	58	3	5.17	1.67	16.04
Both, mother and baby given Nevirapine	404	29	7.18	4.99	10.33
None given Nevirapine	25	6	24.00	10.78	53.42
Duration of breastfeeding					
Not breastfed	164	4	2.44	0.92	6.50
1–26 weeks	240	23	9.58	6.37	14.42
Above 26 weeks	82	11	13.41	7.43	24.22
Mode of delivery					
Vaginal	433	37	8.55	6.19	11.79
Caesarean	54	1	1.85	0.26	13.15
Total	487	38	7.80	5.68	10.72

### Associated factors of MTCT

The results of multivariate competing-risk hazard regression model are given in Table 4. In the multivariate analysis, breastfeeding was a significant risk factor for HIV transmission and the risk was higher for babies who were breastfed for more than 26 weeks (AHR 5.28, *p* = 0.003; 95%CI: 1.75–15.90). Nevirapine administered to both mother and child (AHR:0.25; *p* = 0.002; 95%CI: 0.10–0.61) as well as to either one (AHR: 0.12; p = 0.003; 95% CI: 0.03–0.49) resulted in a significantly reduced risk of MTCT by age 24 months, as compared to infants who were not administrated Nevirapine. In addition, babies born to mothers aged 30 years or more, had a significantly higher (AHR: 6.98; *p* = 0.006; 95%CI: 1.73–28.16) MTCT as compared to babies born to mothers whose age was less than 20 years. In the multivariable model, caesarean section was associated with a non-significant decreased risk of transmission compared with vaginal delivery (AHR 0.20, *p* = 0.125; 95%CI: 0.03–1.56). Mothers who were initiated the ART more than 90 days prior to delivery experienced a higher risk of MTCT by age 24 as compared to mothers who were not initiated on ART (AHR:2.31; *p* = 0.053; 95%CI:0.99–5.38) with a border level of significance. Though not significant, mothers who were initiated on ART within 90 days prior to delivery (AHR:0.65; *p* = 0.486; 95%CI:0.20–2.16) and after the delivery were (AHR:0.44; *p* = 0.342; 95%CI:0.08–2.38) at a lower risk of experiencing MTCT by the age of 24 months.

**Table 4:** Results of competing-risks hazard regression model for vertical transmission of HIV among children by 24 months

Characteristics	Adjusted hazard ratio	*p*-value	95% CI	
Age of mother at pregnancy				
< 20 (Reference)	1.00			
20–24	2.55	0.139	0.74	8.84
25–29	2.30	0.195	0.65	8.12
30+	6.98	0.006	1.73	28.16
Education of mother				
Illiterate (Reference)	1.00			
Primary school	1.02	0.970	0.38	2.70
Above primary school	0.94	0.877	0.40	2.19
Working status of mother				
Not working (Reference)	1.00			
Working	0.87	0.775	0.34	2.23
Know HIV positive status prior to pregnancy				
No (Reference)	1.00			
Yes	0.78	0.594	0.32	1.92
Status of ART initiation by mother				
Not on ART (Reference)	1.00			
Started ART more than 90 days prior to delivery	2.31	0.053	0.99	5.38
Started ART within 90 days prior to delivery	0.65	0.486	0.20	2.16
Started ART after delivery	0.44	0.342	0.08	2.38
Sex of the child				
Girl (Reference)	1.00			
Boy	1.20	0.607	0.60	2.38
Status of Nevirapine administration				
None given Nevirapine (Reference)	1.00			
Both, mother and child given Nevirapine	0.25	0.002	0.10	0.61
Any one given Nevirapine	0.12	0.003	0.03	0.49
Duration of breastfeeding				
Not breastfed (Reference)	1.00			
1–26 weeks	4.34	0.005	1.55	12.16
Above 26 weeks	5.28	0.003	1.75	15.90
Mode of delivery				
Vaginal (Reference)	1.00			
Caesarean	0.20	0.125	0.03	1.56

## Discussion

The advances in the PPTCT program have been one of the major successes in reducing the MTCT rates in India. The present study provides a basis for estimating the burden of Paediatric HIV among HIV exposed children below 24 months of age. The study used a cohort approach and included all maternal HIV exposed children born during the years between 2011 and 2013 who reside in Belgaum district. The study findings are representative of high HIV prevalence (> 1% HIV among ANC from HIV Sentinel Surveillance) districts, within which the coverage of antenatal services, including HIV testing, are at high levels.

Most of the deliveries occurred prior to the introduction of triple drug ART regimen for all pregnant women under the PPTCT programme in India. The study estimated the prevalence of paediatric HIV to be 7.8% in maternally HIV exposed children by age 24 months. Earlier, an overall MTCT rate of 18.6% was identified by age 18 months among HIV exposed children born during 2005–2007 in West Bengal [23]. In a study conducted in Ananthapur district, where all HIV-infected pregnant women were given triple drug antiretroviral therapy (ART) regardless of the CD4 lymphocyte count, the MTCT rate reported was 3.7% [18]. Studies conducted in Delhi and Ahmedabad found an overall MTCT rate of 8.3 and 8.5% [19, 24] among exposed children by age 18 months, respectively, which is close to the results obtained in the present study. The difference in results of MTCT in the studies mentioned above are probably related to the coverage of ART among HIV positive pregnant women. Overall, in our study 51% of pregnant women had been initiated on ART and 42% initiated ART prior to delivery. The studies conducted in Delhi and Ahmedabad reported an ART coverage of 58% and 32%, respectively, among mothers prior to delivery. However, the study in Ananthpur included only the mothers who were on ART prior to delivery.

The study identified three factors; mother’s age above 30 years, breastfeeding and Nevirapine prophylaxis which were found to be significantly associated with MTCT. The results suggest that children born to mothers aged 30 years or more, had 7 times higher risk of MTCT than those who were born to mothers less than 20 years of age. Additionally, the children who are breastfed for more than 26 weeks (equivalent to 6 months) were almost 5 times more likely to be HIV infected by age 24 months of age, than children who are not breastfed. The benefits from the PPTCT programme might have been reversed by prolonged duration of breastfeeding of the HIV exposed children. It is possible that the prolonged duration of breastfeeding also implied mixed feeds. In well-resourced settings, the complete avoidance of breastfeeding has been a major factor in reducing transmission and breastfeeding is not recommended for HIV-infected women [11, 25]. In developing countries like India, owing to the high risk of malnutrition, morbidity and mortality among infants exposed to HIV who are not breastfed, complete avoidance of breastfeeding may not be possible [18]. Thus, in India where prolonged breastfeeding is a cultural norm, the duration of breastfeeding may have to be restricted to less than 6 months, also ensuring that there is no mixed feeding.

Also, there is an urgent need to improve coverage and adherence to ART for HIV-positive mothers, especially during breastfeeding period, in order to reduce the risk of MTCT in postnatal period. The new PPTCT guidelines to provide life-long (triple drug regimen) ART to all pregnant and breastfeeding women living with HIV is effective from January 2014 [2]. Though not significant, we noticed that ART initiation close to the delivery and after delivery was more beneficial in reducing the risk of MTCT. As indicated by earlier research studies [26–28], the present study also indicated that nevirapine prophylaxis administrated to either the child or the mother significantly reduces the MTCT rates. We found that the MTCT rates were 7 times more among children who were not administered nevirapine. However, several studies have demonstrated the selection of nevirapine-resistant HIV-1 variants after single dose nevirapine exposure [29, 30].

The use of ART remains the cornerstone of PMTCT interventions to reduce MTCT rates and research suggests that ART reduces the risk of MTCT [31]. There is an urgent need to improve coverage and adhrence to ART for HIV-positive mothers, especially during delivery and during breastfeeding in order to reduce the risk of MTCT in postnatal period. The present study showed a tendency that mothers who started ART more than 90 days prior to delivery were at higher risk of MTCT than mothers who were not on ART. It is possible that there could be a recall bias or that ART adherence was poor. The ART administration in India was generally based on CD4 count (< 350 cells/mm3), WHO clinical staging and other baseline investigation [32]. It is possible that most of the women who were on ART prior to delivery also had very low CD4 counts at the time of initiation. Adherence to ART regimens is a major challenge among women in both urban and rural areas in India [33]. A recent meta-analysis, suggested that optimal adherence is a challenge during pregnancy and the postpartum period [34]. Virologic and clinical success depend crucially on good adherence [35]. The MTCT rates were found to be lower with high adherence to ART [34]. Though not significant, our study also highlighted the advantage of delivery through caesarean in reducing the MTCT rates.

The study has some limitations. The study used the HIV infected mothers who were tested during the pregnancy in the districts. We could have missed HIV infected mothers who are not tested for HIV. However, program data indicates that 95% of the mothers received at least three or more antenatal checks in the district and more than 85% of all pregnant women in the district had been tested for HIV [36]. However, the HIV transmission rate could be higher among the women who have never even accessed health services and HIV testing during pregnancy or delivery. It is possible that women not accessing health care is low in this district. Other limitations of our study are the lack of data on measurement of ART adherence; objective assessment of exclusive versus mixed breastfeeding and no measurements of viral load among infected women. We did not collect information about the ART regimen that the mothers had been put on, nor did we administer any tool or test to assess ART adherence. Hence, we cannot infer the effect of these factors on our results. Another limitation of the study was that we did not consider potential confounders, such as viral load and the CD4 count of mother in the analysis, since we did not collect this information. A last limitation of our study is that the PPTCT guidelines evolved during the course of the study from single dose Nevirapine for the mother and baby to Triple Drug regimen for mothers. However, since we did not monitor its implementation, this limits our judgement and interpretation of results related to Nevirapine, as the population is not homogenous. Despite the current limitations, our study has pointed to the important finding that the program’s success in reducing MTCT rate is challenged by the breastfeeding practices among HIV infected mothers and this finding has policy implications. These challenges call for limiting exclusive breastfeeding only up to 6 months and not beyond among HIV infected women and other newer effective strategies to promote safe infant feeding among HIV infected mothers in order to bring about a significant reduction in paediatric HIV incidence. The promotion of contraception, especially for women living with HIV above the age of 30 years is important.

## Conclusions

Earlier studies from India were either confined to respondents from few facilities, or assessed HIV incidence among urban dwellers only. The uniqueness of this study is the inclusion of both urban and rural HIV positive mothers from both public and private health facilities in the entire district of Belgaum, that were providing PPTCT services. Most of the studies in India did not use any multivariate statistical techniques. We used appropriate statistical techniques, to control for possible confounders, to determine the risk factors associated with mother-to-child transmission of HIV.

The study suggests that the prolonged duration of breastfeeding of the exposed children has a major implication for the success of PPTCT programme. It calls for appropriate strategies to ensure that WHO recommended infant feeding practices are in place, in order to achieve the zero infection among children [25]. The challenge calls for additional efforts to promote the existing guideline of exclusive breastfeeding only up to 6 months among HIV infected women, if the child is found to be uninfected by that time. Newer effective strategies to promote the practice of safe infant feeding among HIV infected mothers are required in order to end Paediatric HIV infection by MTCT.

## Abbreviations

AHR: Adjusted hazard rate
ART: Anti-retroviral treatment
ARV: Anti-retroviral
CI: Confidence interval
CIR: Cumulative incidence rate
HIV: Human immunodeficiency virus
ICTC: Integrated counselling and testing centres
MTCT: Mother-to-child transmission
PPTCT: Prevention of parent-to-child transmission
